# Gait in ducks (*Anas platyrhynchos*) and chickens (*Gallus gallus*) – similarities in adaptation to high growth rate

**DOI:** 10.1242/bio.018614

**Published:** 2016-07-07

**Authors:** B. M. Duggan, P. M. Hocking, D. N. Clements

**Affiliations:** The Roslin Institute and Royal (Dick) School of Veterinary Studies, University of Edinburgh, Easter Bush, Midlothian EH25 9RG, UK

**Keywords:** Gait, Poultry, Pekin duck, Chicken, Leg health

## Abstract

Genetic selection for increased growth rate and muscle mass in broiler chickens has been accompanied by mobility issues and poor gait. There are concerns that the Pekin duck, which is on a similar selection trajectory (for production traits) to the broiler chicken, may encounter gait problems in the future. In order to understand how gait has been altered by selection, the walking ability of divergent lines of high- and low-growth chickens and ducks was objectively measured using a pressure platform, which recorded various components of their gait. In both species, lines which had been selected for large breast muscle mass moved at a slower velocity and with a greater step width than their lighter conspecifics. These high-growth lines also spent more time supported by two feet in order to improve balance when compared with their lighter, low-growth conspecifics. We demonstrate that chicken and duck lines which have been subjected to intense selection for high growth rates and meat yields have adapted their gait in similar ways. A greater understanding of which components of gait have been altered in selected lines with impaired walking ability may lead to more effective breeding strategies to improve gait in poultry.

## Abstract (precis)

**Summary:** Different bird species bred for meat production have adapted their gait in similar ways to handle the extra loads imposed on their legs by larger muscle masses.

## INTRODUCTION

Intense selection for production traits in poultry over approximately 60 generations has led to considerable genetic gain. During this period the body mass of the meat type (broiler) chicken has increased by 300% (Knowles et al., 2008). One unwanted side effect of this genetic gain has been an increased incidence of locomotion (gait) problems (Paxton et al., 2013). Altered gait in livestock is an important welfare issue, causing a reduction in mobility, that may be associated with pain (McGeown et al., 1999; Danbury et al., 2000; Caplen et al., 2013) and a reduction in normal behaviours (Vestergaard and Sanotra, 1999; Weeks et al., 2000).

Estimates of the prevalence of gait problems in broiler chickens have been reported between 15% and 30% (Kestin et al., 1992; Sanotra et al., 2001, 2003; Knowles et al., 2008). The true prevalence of gait problems is difficult to obtain, because of variation between studies in the strains of birds assessed, the gait scoring systems used, the age at which birds are assessed and the management factors at each site (EFSA Panel on Animal Health and Welfare, 2010). Whereas recent reliable information on the prevalence of leg weakness in poultry is not available, it is widely accepted that the problem causes economic losses for the producer (Yogaratnam, 1995). The scale of gait problems in commercial duck populations is also poorly defined, with the only study (which reported the prevalence of gait abnormalities in 46 flocks of commercial ducks) estimating that 14% of 23-day-old and 21% of 41-day-old Pekin ducks showed signs of gait abnormality (Jones and Dawkins, 2010).

The aetiology of gait problems in poultry is varied and complex. An obvious consequence to selection for high pectoral muscle mass in broiler chickens has been a cranial shift in the body's centre of mass (COM) which has been hypothesised to lead to gait instability related to excess stress on the leg muscles (Corr et al., 2003b; Paxton et al., 2014). Skeletal disorders have also been associated with increased body mass and growth rate, some of which negatively affect gait. These include tibial dyschondroplasia (TD), valgus/varus deformities, bone torsion and bone fractures (Bradshaw et al., 2002). While some of these abnormalities may be painful, others may simply alter gait due to conformational changes (Corr et al., 2003b).

Since gait problems were first reported in broiler chickens (Farm Animal Welfare Council, 1992; Kestin et al., 1992), efforts have been made to alleviate gait issues across various species through selection, with varying results. For example, selection has been shown to reduce the incidence of TD in broilers over the course of two decades (Kapell et al., 2012). However, poor gait still remains; perhaps due to the difficulty in measuring gait and low heritability leading to relatively little genetic gain in the trait (Sandilands et al., 2011). The standard method of gait assessment is a visual gait score (Kestin et al., 1992). While this is a rapid and inexpensive method of high-throughput phenotyping, the visual gait score has been reported to have relatively poor reliability, due to the subjective nature of the score (Kestin et al., 1992; Anon, 2000; Garner et al., 2002). Previous attempts to improve the objectivity of the visual gait score in broilers have led to more reliable estimates (Garner et al., 2002). The development of a better gait score with improved repeatability may lead to better estimates of heritability and long-term genetic gain for gait-related traits in selection programmes. However, objective gait measurement tools used in research, such as kinematic and kinetic systems (Corr et al., 2007; Sandilands et al., 2011; Caplen et al., 2012; Paxton et al., 2013) are unsuitable for use on breeding farms due to costs and time constraints.

The aim of this study was to objectively identify gait changes which have occurred through selection in chicken and duck lines selected for high growth rates and to compare these to conspecifics which have either not been selected for high growth rates (the layer chicken) or which have undergone no artificial selection (the mallard). We also report how certain gait parameters change within lines during growth to slaughter age. Broiler chickens were used as an example of a line selected for high growth rate and layers to represent a line with a growth rate more similar to their ancestral phenotype, the red junglefowl. In Pekin ducks a commercial hybrid and two breeding lines were used as examples of high growth rate birds; these were compared with their ancestral phenotype, the mallard (*Anas platyrhyncos*). The layer chicken and the mallard were assumed to possess an optimal gait for their respective species. It was expected that heavy lines of both species which have undergone selection for increased meat yield would adapt their gait in similar ways to compensate for their change in morphology. A greater understanding of how gait has changed through selection in these lines may inform a more robust gait scoring system based on objective measurement of key gait components and identify which aspects of gait are indicative of the ideal walk.

## RESULTS

Least squares means and standard errors of treatment difference for gait traits in all lines and ages are presented in Table S1.

**Fig. 1.**
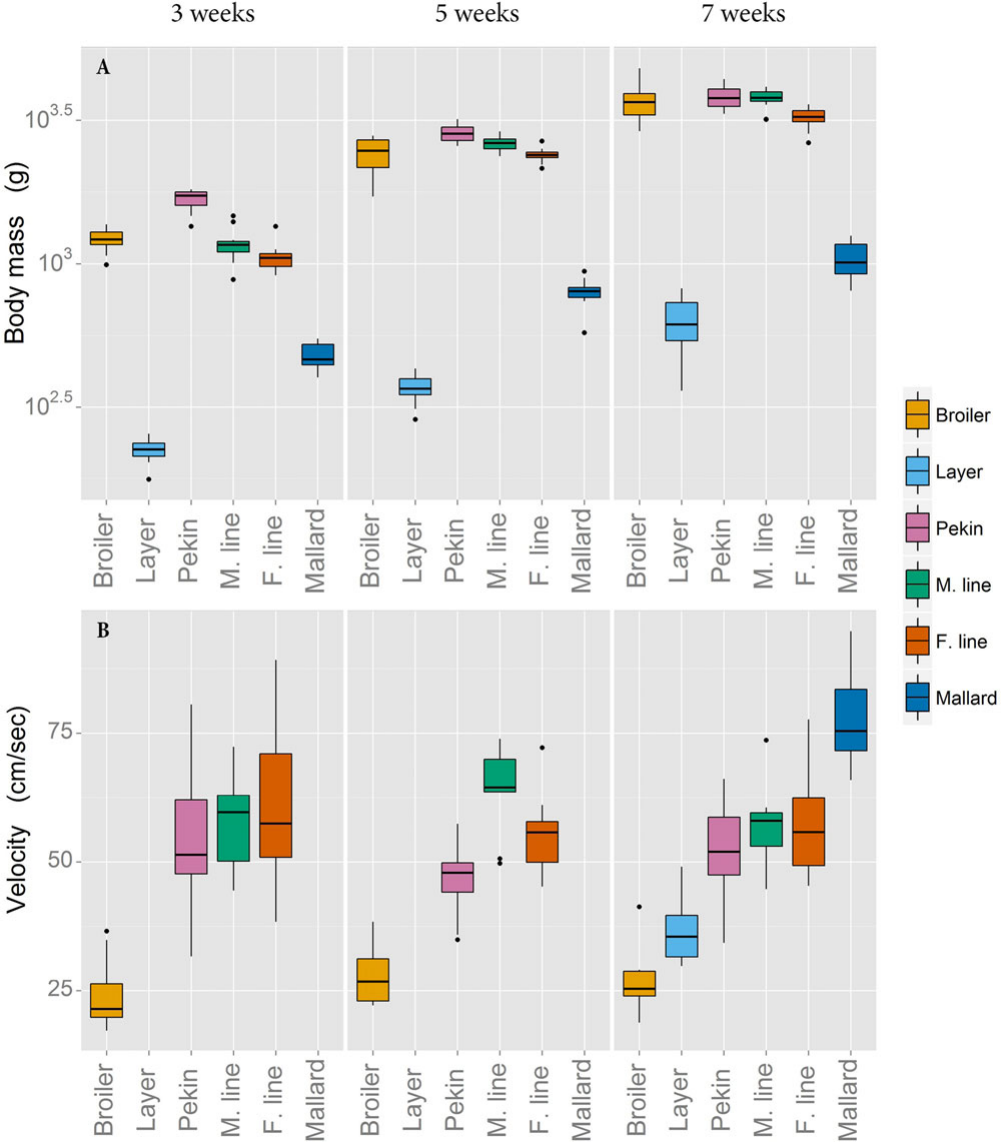
**Body mass and comfortable walking velocity.** Body mass (A) and comfortable walking velocity (B) in the broiler and layer chicken, the Pekin commercial hybrid (Pekin), the Pekin male breeding line (M. Line), the Pekin female breeding line (F. line) and the mallard. Body mass is presented on a log scale for clarity. For velocity, each value represents the mean velocity of five walks from a single bird. Velocities of the layer chicken and mallard were not recorded at three and five weeks of age due to limited sensitivity of the pressure walkway at these body masses.

**Fig. 2.**
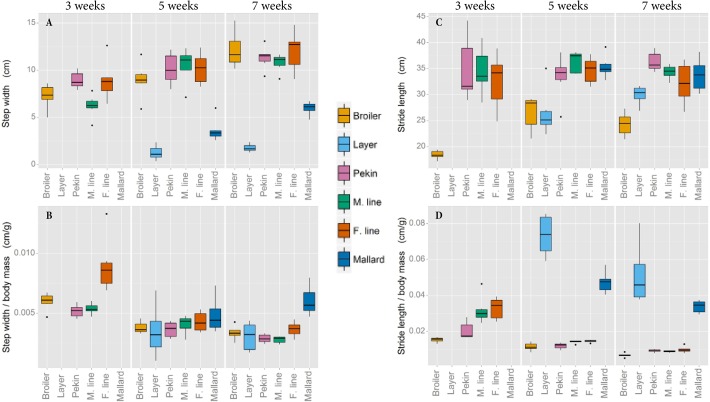
**Step width and stride length.** Step width and stride length are presented as raw means (A and C, respectively) and as ratios of body mass (B and D, respectively). Data was not recorded at three weeks of age in the layer chicken or mallard line due to limited sensitivity of the pressure walkway at this body mass.

The divergence in growth rate and body mass between lines selected for carcass traits and ‘unselected’ lines is displayed in Fig. 1A. Fig. 1B shows the comfortable velocity ranges of each line, at three, five and seven weeks of age. The layer chicken moved at a significantly faster speed than the broiler and the Pekin commercial hybrid walked significantly faster than both chicken lines (*P*<0.005). In Experiment 2, the mallard walked significantly faster than the Pekin breeding lines (*P*<0.001). In each species, the lines unselected for high muscle mass (the layer chicken and mallard) both walked with a significantly narrower step width than their heavier conspecifics (the broiler chicken and Pekin duck respectively) (*P*<0.001). Both body mass and the length of the tibiotarsus (a proxy for leg length) were initially included as covariates in the analysis of step width but had no effect. There was a line by age interaction in Experiment 1 (*P*=0.021); step width increased substantially after five weeks in both chicken lines whereas no substantial increase in step width was observed after five weeks in the Pekin commercial hybrid (Fig. 2A). The ratio of step width to body mass is presented in Fig. 2B. In Experiment 1, the stride length differed between lines (*P*<0.001). Tibiotarsal length and body mass were included in an initial statistical model as covariates but had no effect. The layer had a longer stride than the Pekin hybrid, which had a longer stride than the broiler chicken (*P*<0.001). There was a line by age interaction (*P*=0.012); broiler stride length decreased after five weeks but the stride lengths of the layer and Pekin hybrid increased. In Experiment 2, there was no difference in stride length between duck lines (Fig. 2C). The ratio of stride length to body mass is presented in Fig. 2D.

**Fig. 3.**
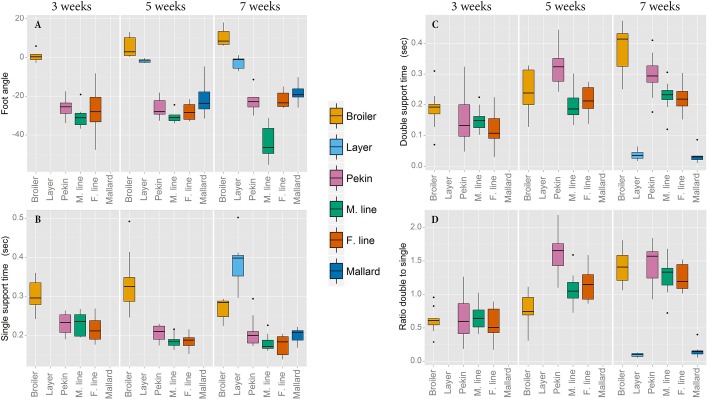
**Foot angle, single foot support time, double foot support time and the ratio of double to single foot support time at three five and seven weeks.** (A) A positive foot angle represents externally rotated feet while a negative value indicated internal rotation. (B) Single support time is the proportion of the gait cycle during which the bird has only one foot in contact with the ground. (C) Double support time is the proportion of the gait cycle during which both feet are in contact with the ground. (D) Ratio of double to single foot support time. Foot angle data for the layer chicken and mallard at three weeks of age and support time data for the same lines at three and five weeks of age were omitted due to limited sensitivity of the pressure walkway at these body masses.

The angles at which the feet were placed during walking were significantly different between broiler and layer chickens in Experiment 1 (Fig. 3A); layers' feet aligned sagittally to the direction of travel whereas those of broilers were externally rotated (*P*<0.001). The feet of the Pekin commercial hybrid were internally rotated compared with both chicken lines (*P*<0.001). In Experiment 2 there was no difference in foot angle between the mallard and the female Pekin breeding line (which both displayed similar means and variation of foot angle to the Pekin commercial cross in Experiment 1); however the foot angle of the male Pekin line was more internally rotated (*P*=0.001) compared with the female line and the mallard (Fig. 3A). The foot became more internally rotated after 5 weeks of age in the male Pekin line whereas the feet of the mallard and female line both became less internally rotated after this age, leading to a line by age interaction (*P*=0.008).

In both species, heavy lines spent more time being supported by two legs during walking when velocity was accounted for as a covariate (*P*<0.001) (Fig. 3C). The ratio of double support time to single support time (with walking velocity accounted for as a covariate) was greater in heavier lines of both species compared to their lighter conspecifics (*P*<0.001) (Fig. 3D).

**Fig. 4.**
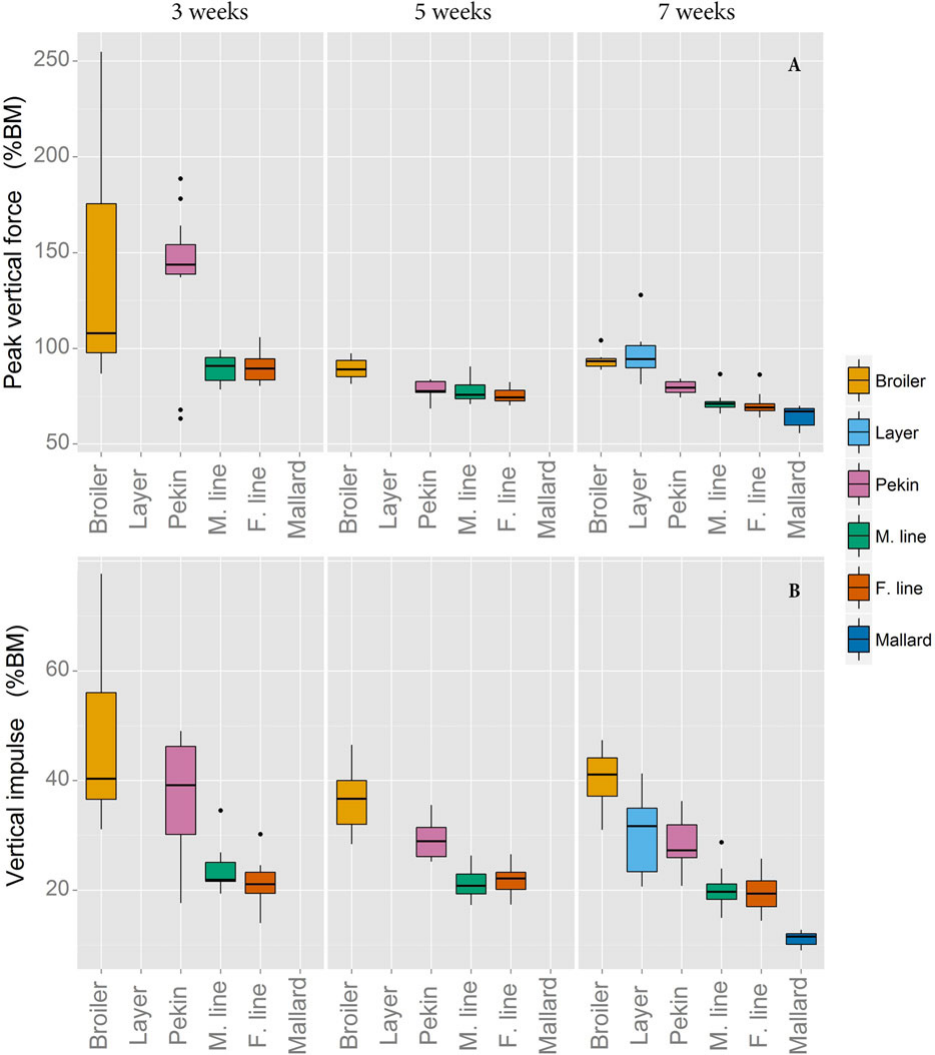
**Mean peak vertical forces and vertical impulse forces.** Mean peak vertical forces (A) and vertical impulse (B) values expressed as a percentage of body mass. Data for the layer chicken and mallard at three and five weeks of age were omitted due to limited sensitivity of the pressure walkway at these body masses.

No differences were found in PVF between the lines in Experiment 1 (Fig. 4A). However, PVF significantly decreased with age (*P*<0.001). In Experiment 2 PVF was also found to decrease with age (*P*<0.001). Significant differences were seen between lines in Experiment 2; the male and female Pekin lines produced higher PVFs than the mallard (*P*<0.005). Vertical impulse (Fig. 4B) was greater in the broiler chicken than in both the layer chicken and Pekin commercial hybrid (*P*<0.01). In Experiment 2 both the male and female Pekin lines produced a higher vertical impulse than the mallard (*P*<0.001). In both experiments vertical impulse increased with age (*P*<0.001 in Experiment 1 and *P*=0.029 in Experiment 2).

**Fig. 5.**
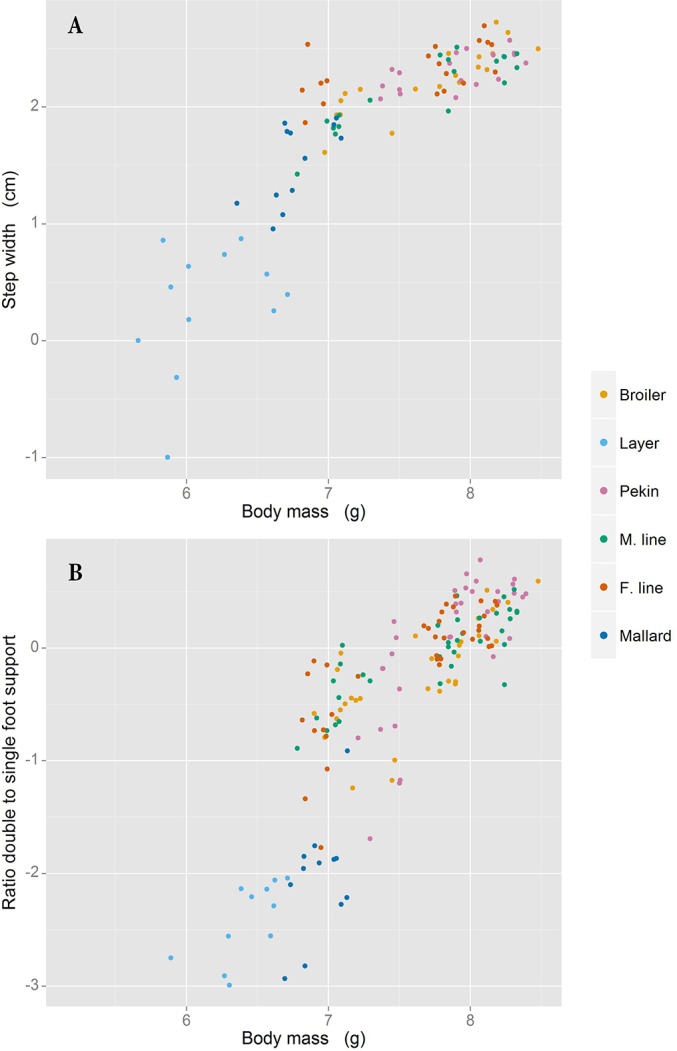
**Step width and foot support time ratio versus body mass.** Step width (A) and the ratio of double foot support time to single foot support time (B) regressed against body mass. All values are logged. Data at all ages is included; however data for the layer chicken and mallard at three weeks of age were omitted due to limited sensitivity of the pressure walkway at these body masses.

Step width changed with both positive allometry and isometry, depending on line (Table 1). No relationship with body mass was observed with stride length, with the exception of the broiler chicken; in this line, stride length scaled isometrically. The ratio of double to single foot support time scaled with positive allometery in all lines, with the exception of the mallard, in which no relationship with body mass was observed for this trait. The allometry of both step width and the ratio of double to single foot support time are presented for all lines in Fig. 5A and B respectively.

**Table 1.**
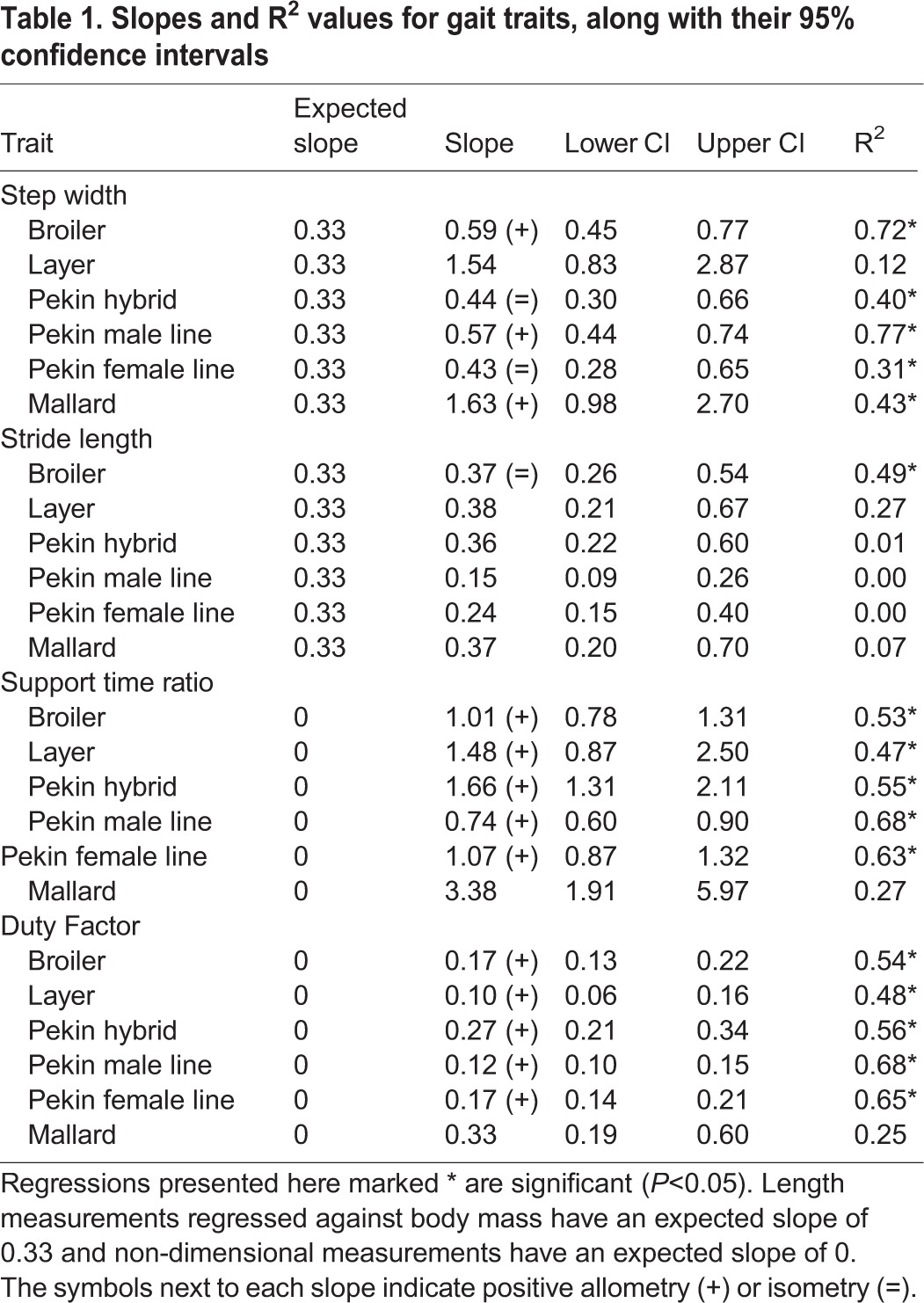
**Slopes and R^2^ values for gait traits, along with their 95% confidence intervals**

## DISCUSSION

These results demonstrate that gait variables, both within and between bird species, change throughout growth. They also highlight the effects that intense selection for rapid growth has had on the gait of modern broiler chickens and ducks.

### Velocity

The measures of velocity used in this study represent the birds' comfortable walking speeds. The speed recorded from each bird is an average of the five walks that were closest to each other in velocity. The ‘preferred’ walking speeds of duck lines were greater than those of chickens. Also, within each species, lines selected for high growth rate and meat yield were slower than their ‘unselected’ conspecifics (Fig. 1B).

The fact that chickens do not walk as quickly as ducks is not unexpected – ducks undergo relatively early leg development, reaching adult leg size by five weeks of age, whereas the legs of chickens continue to grow in size after slaughter age at seven weeks (Dial and Carrier, 2012; Duggan et al., 2015). Therefore it is unsurprising that ducks find it easier to achieve higher walking speeds compared with chickens. Why layer chickens and mallards, both of which have not been selected for high growth rate, reach faster walking speeds than their much larger conspecifics is less obvious. The markedly different hindlimb architecture of the heavier lines combined with a cranial shift in the body's COM due to a disproportionally large increase in breast muscle may have led to an imbalanced gait which requires lower speeds (and higher duty factors) to improve stability. These differences in body morphology have been highlighted as a cause for altered gait in broiler chickens (Corr et al., 2003b; Paxton et al., 2013).

Velocity is not just a measure of an individual's ability to move at a certain speed but also their motivation. We found that each bird had a range of speeds at which it could move. The differences in birds’ preferred velocity between lines was relatively large and the variability in velocity within lines was relatively small, suggesting that these values are not just an indication of individual motivation (which would create large variation within lines) but are more likely a result of morphological differences which confine each line to a limited range of ‘comfortable’ walking speeds.

### Step width and stride length

Step width was greater in heavier lines compared with their lighter conspecifics (Fig. 2A). It is expected that a wider step width, while providing a good base of support during standing, will lead to poor balance during walking. If the stance is wide when the swing leg is lifted during walking then the body's COM will not be aligned with the centre of pressure of the supporting foot. The COM will begin to move away from the supporting leg until the swing leg is grounded to provide stability once again. This process leads to a large lateral movement of the body's COM during walking, which may be energetically expensive and could lead to stumbling. The necessity to ground the swing leg quickly to ensure the COM does not fall to the ground potentially explains why the heavy broiler chickens have a shorter stride than the layers; however the Pekin lines and the mallard have a similar stride length (Fig. 2C). The mallard's step width (when expressed as a ratio of body mass), although narrower than the heavier Pekin is relatively wider than other lines (Fig. 2B), perhaps due to its naturally wide hull-shaped body. This may explain the lower than expected stride lengths observed in the ancestral line. A relatively shorter stride length (Fig. 2D) may contribute to the lower velocities seen in these heavy lines compared with the lighter lines. Conversely, in the layer chicken and mallard lines, the step width is narrower and the body's COM is closer to the vertical axis of the supporting foot during walking. This balanced support allows these slow-growth lines to spend less time supported by both feet during walking as their COM is relatively stable above a single supporting foot.

### Ratio of double to single foot support

The ratio of double foot support to single foot support is a temporal measure of limb placement which may be an indicator of balanced gait, as suggested by Corr et al. (2003b). Theoretically, a bird with an imbalanced gait will spend more time supporting its weight across both feet; therefore, a low ratio of double to single foot support is indicative of a bird with a well-balanced gait. The current data show that, at seven weeks of age, both layer chickens and mallards have much lower ratios of double to single foot support than their heavier conspecifics, suggesting that these lines, which are unselected for high muscle mass, have better balanced gaits (Fig. 3D). This lack of balance in high growth lines may result from an increase in pectoral muscle mass which has led to a cranial shift in the COM of broiler chickens (Corr et al., 2003a,b; Paxton et al., 2014). While it was not possible to measure temporal foot placement in layers and mallards at three and five weeks of age, data from the high-growth lines also suggests that younger (lighter) birds, which have less pectoral muscle mass, have lower ratios of double to single foot support and thus are better balanced when walking. Also, for heavier birds, the greatest period of stress on the leg bones occurs when the entire body mass is supported through one foot; distributing body mass across both feet by increasing double foot support time would reduce the likelihood of bone damage, as suggested by Caplen et al. (2012).

### Foot angle

The angle at which the foot is placed during the stance phase of walking can affect balance by moving the base of support to a position either more or less medially aligned with the body's COM. In seven week old broilers, the feet are externally rotated (pointing outwards). This has previously been reported in heavy broiler chickens (Corr et al., 2003b). Theoretically, this would allow the middle toe to extend laterally away from the body's COM, thus providing a wider base of support, extending the ‘safe zone’ in which the body's COM can move laterally out of alignment with the supporting foot without causing instability. This is important as broilers shift their COM laterally while walking to ensure that the COM is medially aligned with the supporting foot before lifting the swing foot (Corr et al., 2003b). Turkeys employ a similar movement (Abourachid, 1991). In contrast, the feet of all duck lines were internally rotated (Fig. 3A). In theory, pointing the toes inward would partially counteract the wide stance seen in heavy lines, which leads to shorter stride lengths and hence lower velocities. An internally rotated foot position would align the toe more medially to the body's COM, improving stability during single foot support, but also reducing the safe zone in which the COM can move without causing instability during walking. That this internal foot rotation is also seen in the mallard suggests that this trait has not developed due to rapid growth or increased body mass but rather is an adaptive trait in the wild phenotype. By seven weeks, the distal end of the tibiotarsus has rotated internally (Duggan et al., 2015), and this may partially explain foot placement in ducks. However, previous studies in broiler chickens have found limited evidence for a link between bone torsion and foot rotation (Corr et al., 2003a,b). It is not clear why the feet of the male Pekin line are rotated internally to a much greater extent than the other duck lines. It is possible that torsion of the tarsometatarsus, as has been observed to occur in the broiler chicken (Duff and Thorp, 1985) may play a role. Subjectively, the male Pekin line did not display noticeably worse gait than the other Pekin lines.

### Peak vertical force and impulse

Mean peak vertical forces and vertical impulses applied through the ground during walking are plotted in Fig. 4, where both are expressed as a percentage of body mass. While pressure platform systems are generally known to provide different values of forces compared to measurements made by force plates, the values are reliable to use for comparisons between individual animals (Lascelles et al., 2006). The lighter lines used for this study were of a mass which was close to the limits of detection for this pressure walkway and the data from three and five week layer chickens and mallards for certain traits were not analysed. At seven weeks of age, layers and broilers did not differ in the peak vertical forces (expressed as a percentage of body mass) they applied through the ground when walking (Fig. 4A). However, mallards at this age produced lower peak vertical forces (as a percentage of body mass) than the heavier Pekin lines. In commercial lines relative peak vertical forces decreased as the birds grew. At three weeks, broiler chickens and Pekin ducks can subjectively be described as having clumsy gaits. Neural control of foot placement and leg muscle function may not be fully developed at this age and it is possible that rapid leg acceleration is responsible for these higher ground reaction forces in certain younger birds. Birds at this age are growing rapidly and these allometric changes may lead to difficulty judging both distances of anatomical points in relation to the rest of the body and muscle force output (Carrier, 1996). The large variation in peak vertical force values observed in the broiler chicken and Pekin commercial cross at three weeks suggests that some birds are maturing earlier than others; some early maturing birds may have already developed more complete neural control of leg movements by this age and so may not display large ground reaction forces. Any interpretations of peak vertical force measurements should take into account the sampling frequency, which in this study was 62.5 Hz. A higher frequency allows more accurate determination of peak vertical force events. During this study it was not possible to measure at a higher frequency; memory restrictions dictated that lower sampling frequency be used in order to capture information on each birds entire walk. While this frequency was considered to be adequate for birds walking at this pace, the possibility remains that some peak vertical force events may not have been detected.

Vertical impulse (force, as a percentage of body mass, applied across time) values do not change as the birds age (Fig. 4B). Although the peak vertical forces (as a percentage of body mass) do not change between five and seven weeks, the actual peak force acting on the bones is increasing, because body mass is increasing during this time. As the greatest stress on the leg bones occurs during single foot support, it is possible that, as birds grow heavier, they increase double foot support time to counteract these increases in peak vertical forces and thus a constant vertical impulse is maintained. At seven weeks, the smaller layer chicken and mallard lines produce lower vertical impulses than their heavier conspecifics, most likely due to lower double foot support times in the gait of layers and mallards. The relatively large values observed in the layer line are most likely the result of the high peak vertical forces produced by these birds (Fig. 4A).

### Allometry

The ratio of double foot support time to single foot support time and duty factor both scaled with positive allometry for all lines except the mallard, for which no relationship with body mass was observed. Duty factor is another way of expressing the double to single support time ratio and so it is expected that the two scale with a similar allometry (although the scaling exponent of each trait differs as one is a proportion and the other a ratio). When the entire mass of the bird is supported by one leg (during single support) the strain on the leg bones is likely to be at its greatest and the heavy lines which have a wide step width are likely to be unbalanced. Increasing the double support time alleviates the impact of these issues on mobility. As birds become heavier it is possible that they increase their double support time above the lower limit that is required to prevent them becoming unbalanced, which in turn leads to positive allometry as observed in these traits. Step width scaled either with isometry or with positive allometry in different lines whereas stride length did not scale to body mass.

### Conclusions

Intense selection for economic traits such as breast muscle mass and growth rate have been accompanied by dramatic changes in several components of gait in both chickens and ducks. The heavy lines of both species have diverged to a similar extent from their ‘unselected’ conspecifics for certain gait traits, suggesting the use of similar strategies to deal with instability due to increased growth or breast muscle mass. Certain traits, such as foot angle, also differ between ‘unselected’ lines, indicating different evolutionary pressures acted on these species prior to domestication. These data can be used to improve the objectivity of gait scoring: by focusing on certain gait components which are likely to play a key role in balanced gait (such as step width or stride length), it may be possible to improve heritability estimates for gait traits and increase selection success.

## MATERIALS AND METHODS

### Animals and husbandry

The gait of 216 birds of different lines was measured objectively using a pressure-sensitive walkway (Tekscan Animal Walkway, Tekscan, Boston, USA) at three ages in two separate experiments; each experiment used different lines of birds. During the first experiment 36 broiler chickens (Ross 308), 36 layer chickens (Lohman Brown) and 36 Pekin ducks (Cherry Valley commercial hybrid) were raised in walled research pens. The second experiment used the same pens to house 36 heavy male line Pekin ducks, 36 lighter female line Pekin ducks (both Cherry Valley breeding stock) and 36 mallards (Hy-Fly Game Hatcheries, UK). Alongside general health and reproductive traits, the male Pekin line is selected with a greater emphasis on feed efficiency whereas the female Pekin line is selected with a greater emphasis on reproductive traits. These Pekin lines were chosen because they are representative of the breeding stock, which is the target group for improving gait by genetic selection. Both these duck breeding lines contained equal numbers of males and females.

Birds were raised following industry guidelines as much as possible. All birds were initially housed from day of hatch under brooder lamps in a single pen per line to regulate temperature. At seven days, birds were randomly allocated in a randomised block design to two blocks of nine pens. Each pen (2.16 m^2^) contained four males and four females in an area of 0.27 m^2^ per bird, increasing to 0.36 m^2^ per bird from 21 days and 0.54 m^2^ per bird from 35 days as birds were removed for measurement. The lighting regime was 23 h light: 1 h dark at hatch, reducing by one hour light per day for the first seven days and remaining at 16 h light: 8 h dark thereafter. The mean light intensity in each pen was 120 lux. Barn temperature was 25°C at two weeks, reducing to 24°C at three weeks, 22°C at four weeks and remained at 20°C from five weeks until termination. Experiment 1 used wood shavings as a substrate as this is the industry norm for chickens. Experiment 2 used straw as a substrate, as is the case on most UK duck farms. All birds were fed *ad libitum* and water was provided *ad libitum* in suspended bell drinkers. Broilers were given a commercial starter feed for the first 10 days, grower from day 11-35 and finisher from day 36 onwards. Layers were fed on a commercial starter feed for the first 35 days before transferring to a grower feed from day 36 onwards. All duck lines in both experiments were fed on a starter feed until day 10 and on a grower feed thereafter: both duck diets were supplied by the breeding company.

The study was approved by the Veterinary Ethical Review Committee at the University of Edinburgh.

### Data collection

At three ages (21, 35 and 49 days) two randomly selected birds (one male and one female) from each pen (six males and six females per line) were walked repeatedly over a Tekscan pressure walkway (Tekscan, Boston, MA, USA). The walkway consisted of two sensing tiles connected together to form a single low-profile 1 m×0.5 m pressure walkway which recorded at a resolution of 1.4 sensing elements per cm^2^. Two ‘Tekscan EH-2 Evolution’ handles were used to connect this system to a laptop computer, allowing kinetic data to be analysed using proprietary software (Tekscan Walkway, v7.02). The walkway was calibrated as per manufacturer guidelines, using pressures which were appropriate for the weight of the birds to be recorded. Proprietary equilibration files (10 PSI and 20 PSI) were used when gathering data. In order to capture information on the entire walk of each bird, the pressure walkway recorded at a frequency of 62.5 Hz. This sampling frequency, while lower than usual for studies of this kind, was necessary due to memory restrictions of the software. Birds were motivated to walk in a straight line over the pressure walkway by placing 50 cm high plywood boards on each side parallel to the walkway. The walkway was covered by a 1 mm thick latex sheet to ensure the birds did not slip. Each bird was released at one end of the walkway and allowed to walk freely (away from the camera) towards two pen-mates which were held in a pen at the other end of the walkway. As a standardisation check, each walk was recorded using a video camera (Microsoft LifeCam Studio), which linked simultaneously to the pressure data collected by the Tekscan software. At least 12 ‘satisfactory’ walks were recorded for each bird. A walk was deemed satisfactory if the bird moved at a steady pace in a straight line without slipping or stumbling. Birds were allowed to walk at their own preferred speed. After 12 walks had been recorded, each bird was euthanatised and dissected to assess leg morphology (Duggan et al., 2015). The data from each walk was analysed using Tekscan software. Each walk was checked again for pausing, stumbling and straightness by viewing the recorded video clips, which afforded an alternate view (from behind, at the level of the birds' head). Any walks which did not capture four successive steps in a straight line on the recording area of the pressure platform, or which showed pausing/stumbling on video, were discarded. A custom script (Python) was used for the remaining walks of each bird to select the five walks which deviated least in velocity. An ‘ideal’ velocity for all birds was not chosen as birds differed in their average velocity depending on age, line and behavioural traits such as shyness or fear and because forcing animals to walk at a particular speed may lead to inconsistent gaits as has been observed in other species (Voss et al., 2010). The five walks which deviated least in their velocity were considered to be most representative of each bird's comfortable walking speed. Data from these five walks were averaged for each bird to obtain measures of velocity, step width, stride length, foot angle (whether the middle, third, toe is internally or externally rotated during ground contact), peak vertical force (PVF, the force applied through the ground during stance time), vertical impulse (a product of the vertical force and the time over which it is applied), support time (the time spent supported by either a single foot or both feet) and duty factor (the proportion of a single gait cycle during which one foot is in contact with the ground). Step width is the distance between the lines of progression of the left and right feet. The line of progression of each foot was determined by drawing a line from the point most posterior to the middle toe of the foot for consecutive steps of that foot. Stride length is the distance measured parallel to the line of progression of a foot, between the posterior heel points of two consecutive footprints of the foot in question. Although 12 birds from each line at each age were walked over the pressure walkway, only seven broiler chickens at seven weeks of age were capable of displaying ‘normal’ gait. Gait data from the remaining five ‘lame’ broilers were not included in the analysis at this age.

### Analysis

Gait data collected by the pressure platform were analysed by a split-plot statistical model using restricted maximum likelihood (REML), with effects for pen nested within block and treatment effects of genetic line, age and sex. The resulting variance components were used to ascertain differences between each line by *t*-test (at a significance level of *P*<0.01). Certain traits (step width, stride length and foot angle were measured manually from the trace of foot pressures left on the walkway to avoid measurement errors from the proprietary software's automated measuring system due to its inability to correctly identify the foot pressure pattern consistently. As manual measurement of these traits is labour intensive, six birds from each line at each age (with the exception of 3-week-old layers and mallards) were selected randomly for measurement. Because birds were selected randomly for these measurements, blocking effects were not included in the statistical model for analysing these traits. Separate REMLs were performed to compare the lines from Experiment 1 (the broiler chicken, the layer chicken and the Pekin commercial hybrid) and the lines from Experiment 2 (the male Pekin line, the female Pekin line and the mallard).

Single support time, double support time and the ratio of double to single support time were analysed by general ANOVA as these traits were only measured at one age (seven weeks) when layer chickens and mallards provided large enough pressures for accurate measurement of these traits. Tukey post hoc tests (at a significance level of *P*<0.01) were performed to ascertain differences between lines.

Certain gait traits were also analysed allometrically by assessing their scaling relationships with body mass; the log of each trait was regressed against the log of body mass using a reduced major axis regression. The slope of the resulting regression equation was compared to the expected scaling component for that trait. Length measurements were expected to scale to body mass^0.33^ and non-dimensional traits (such as duty factor) were expected to scale to body mass^0^.
